# Evaluating the Performance of LYDIA: An AI-Powered Assistant in the Detection of Metastatic Tumors to Optimize Clinical Workflows and Inform Soft Tissue Surgical Decision-Making

**DOI:** 10.3390/bioengineering13091021

**Published:** 2026-09-01

**Authors:** Georgios Eleftherios Kalykakis, Isaak Tarampoulous, Athanasia Sepsa, Giorgos Agrogiannis, Giannis Vamvakaris, Menelaos G. Samaras, Christos Spyropoulos, Chrysostomos Manolis, Nikolaos Niotis, Thomas Papathymiopoylos, Konstantinos N. Vougas

**Affiliations:** 1 DeepPath PC, N.Psychiko, 15451 Athens, Greece; 2 1st Department of Pathology, Faculty of Medicine, National and Kapodistrian University of Athens, 11527 Athens, Greece; 3 Department of Pathology, Sotiria Chest Diseases Hospital, 11527 Athens, Greece; 4 2nd Department of Pathology, Medical School, National and Kapodistrian University of Athens, Attikon University Hospital, 12462 Athens, Greece; 5 Proteomics Research Unit, Biomedical Research Foundation of the Academy of Athens, 11527 Athens, Greece

**Keywords:** digital pathology, artificial intelligence (AI), metastasis detection, whole slide imaging (WSI), diagnostic accuracy, workflow efficiency, cost–benefit analysis, soft tissue reconstruction, soft tissue repair

## Abstract

The integration of artificial intelligence (AI) into digital histopathology has the potential to improve the accuracy and efficiency of metastatic cancer diagnosis. We evaluated LYDIA (LYmph noDe assIstAnt), an AI-based decision-support system, for both standalone diagnostic performance and its impact on histopathologists’ workflow, diagnostic accuracy, and resource utilization. LYDIA was evaluated on a blinded dataset of 366 whole-slide images (WSIs) from breast, colorectal, lung, and skin cancers. Standalone performance demonstrated excellent discrimination, achieving ROC-AUC values of 0.995, 0.963, 0.973, and 0.983 for breast, colorectal, lung, and skin cancers, respectively. Clinical utility was further assessed in a multi-reader study involving four experienced histopathologists interpreting 105 WSIs with and without AI assistance. AI-assisted diagnosis significantly reduced time-to-diagnosis across all metastasis sizes, with a maximum 1.59-fold acceleration for micro-metastases, corresponding to a mean time saving of 26.5 s per WSI. LYDIA also improved diagnostic sensitivity from 77.3% to 87.3%. These findings demonstrate that LYDIA can enhance both the efficiency and accuracy of lymph node metastasis detection while reducing diagnostic workload and the need for ancillary testing. Beyond improving routine pathology workflows, the system’s rapid inference capabilities and human-expert level performance may support future intraoperative diagnostic applications, enabling timely and automatic or semi-automatic assessment of nodal status to inform surgical decision-making. The resulting reductions in diagnostic time and ancillary testing costs have the potential to improve healthcare resource utilization and patient care, mainly in soft tissue reconstruction and surgical repair decisions.

## 1. Introduction

Histopathology is central to diagnosing metastatic cancer through the assessment of lymph nodes collected during primary tumor removal or biopsy [1]. Recent advances in slide scanning technology have transformed traditional microscopic evaluation, enabling histopathologists to assess digitized slides [2,3]. Digital pathology opened opportunities for AI to provide decision-support for various diagnostic tasks including tumor detection on lymph nodes (a high-volume task) to accelerate diagnostics. Deep learning models show high precision in image analysis, sparking interest in their use in pathology [4,5]. Yet, clinical adoption remains limited due to concerns around trust and AI displacing pathologists [6]. However, the utility of AI as a decision-support tool rather than a substitute in pathology is unclear. Thus, comprehensive evaluations across tumor types and real-world settings are needed to demonstrate utility and workflow efficiency [7,8].

**Figure 1:**
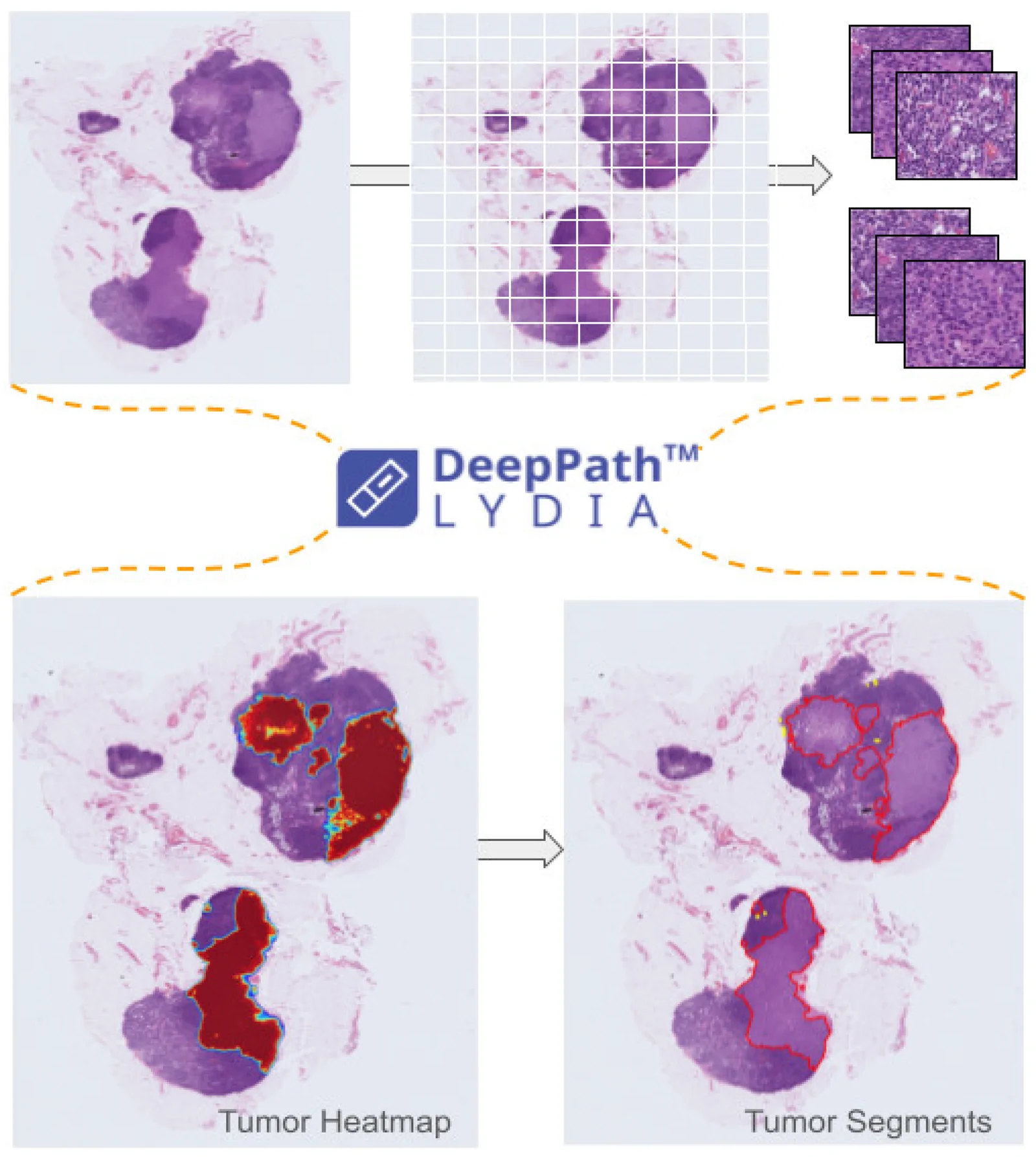
**Overview of the LYDIA pipeline.** WSIs of H&E-stained lymph nodes are divided into patches and a probability of tumor presence is assigned to each patch. Patch-level predictions are aggregated into tumor heatmaps, which are refined into discrete tumor segments and returned to the user for review.

DeepPath has developed LYDIA (LYmph noDe assIstAnt), a commercially available AI-powered decision support system designed to detect tumors in lymph node whole-slide images (WSIs) (https://www.deeppath.io/deeppath-lydia/ accessed on 29 July 2026). LYDIA processes H&E-stained WSIs across multiple formats and resolutions, identifying tumor-containing regions of interest (ROIs) that are presented to histopathologists as heatmap overlays highlighting tumor locations (Figure 1), enabling faster and more accurate diagnosis. Previously published AI-based models have demonstrated the feasibility of detecting lymph node metastases, but they present notable methodological and clinical limitations. Landmark studies, including the CAMELYON challenges [9] and the works of Steiner et al. (2018) [10] and van Dooijeweert et al. (2024) [11] for lymph nodes and Campanella et al., 2019 [7] for the prostate, have relied largely on traditional convolutional neural networks (CNNs), which can struggle with morphological generalization across varied tissue types. These implementations have also focused primarily on single-site cancers. LYDIA addresses these gaps through two key advances. Methodologically, it leverages NeoPath, a lightweight (22M-parameter), self-supervised Vision Transformer (ViT) foundation model [12]. Clinically, it extends beyond existing systems by offering robust pan-cancer detection that beats the competition [13]—validated across breast, colon, lung, vulvar, head and neck, and skin cancers—with a highly optimized architecture that achieves 3 min inference times. This rapid turnaround positions LYDIA not only for routine post-operative pathology review but also as a potential real-time intraoperative decision-support tool.

This study evaluates LYDIA’s standalone performance on a blinded external dataset, as well as its impact on diagnostic speed and accuracy when used for decision-support in a retrospective multi-reader assessment conducted under clinical-like conditions. Because more accurate diagnosis reduces the need for follow-up immunohistochemistry requests [11], we further assess the associated potential for cost savings. Studies establishing the performance, usability, and tangible benefits of AI decision-support tools—for both patients and the broader health system—remain essential to building the evidence base required to justify their adoption into routine clinical workflows. In addition to enhancing routine pathology workflows, the system’s rapid inference capabilities and expert-level performance may enable future intraoperative diagnostic applications, facilitating timely AI-powered evaluation of nodal status to support soft-tissue surgical decision-making. By reducing diagnostic turnaround times and the need for ancillary testing, the system has the potential to improve healthcare efficiency and enhance patient care.

## 2. Materials and Methods

### 2.1. Computational Frameworks

AI/ML model development was implemented in Python (3.11.0) using the PyTorch (1.13.0) [14] deep learning framework. The Dask framework (2022.12.0) [15] was utilized for enabling efficient parallelized processing of large-scale datasets. Statistical analyses and figure generation were performed in R (4.2.2) [16].

### 2.2. Model-Training Datasets

For model-training, 15,380 WSIs were used. The number of WSIs stratified across tissue types is presented in Table 1. The model-training dataset consisted of public and in-house sources. The public sources were the CAMELYON dataset (breast—n = 374) [9], GDC dataset (multiple tissue types—n = 3181) [17], TissueNet dataset (cervix—n = 585) [18] and the PANDAS dataset (prostate—n = 9999) [19]. The in-house dataset was sourced from the Manchester Cancer Research Center and consisted of lymph nodes from patients with breast cancer (n = 141), colon cancer (n = 372), other gastrointestinal cancers (n = 269), lung cancer (n = 216), and melanoma (n = 243).

### 2.3. Study Cohorts

**Figure 2:**
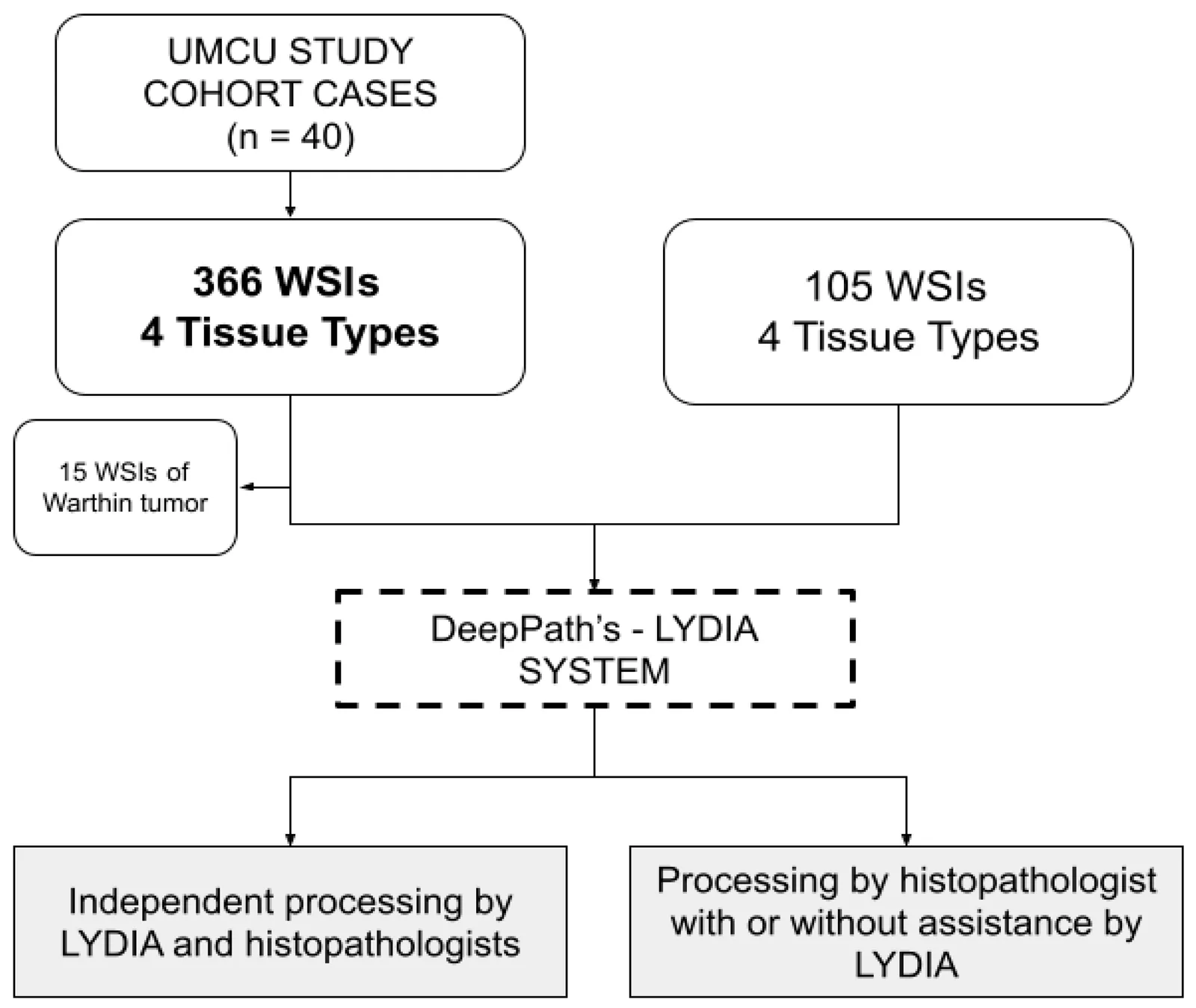
**Overview of the two study cohorts used to evaluate LYDIA.** The external UMCU cohort (351 WSIs) was used for system performance validation, while the retrospective multi-reader assessment cohort of 105 WSIs assessed diagnostic speed and accuracy with and without AI assistance.

**Figure 3:**
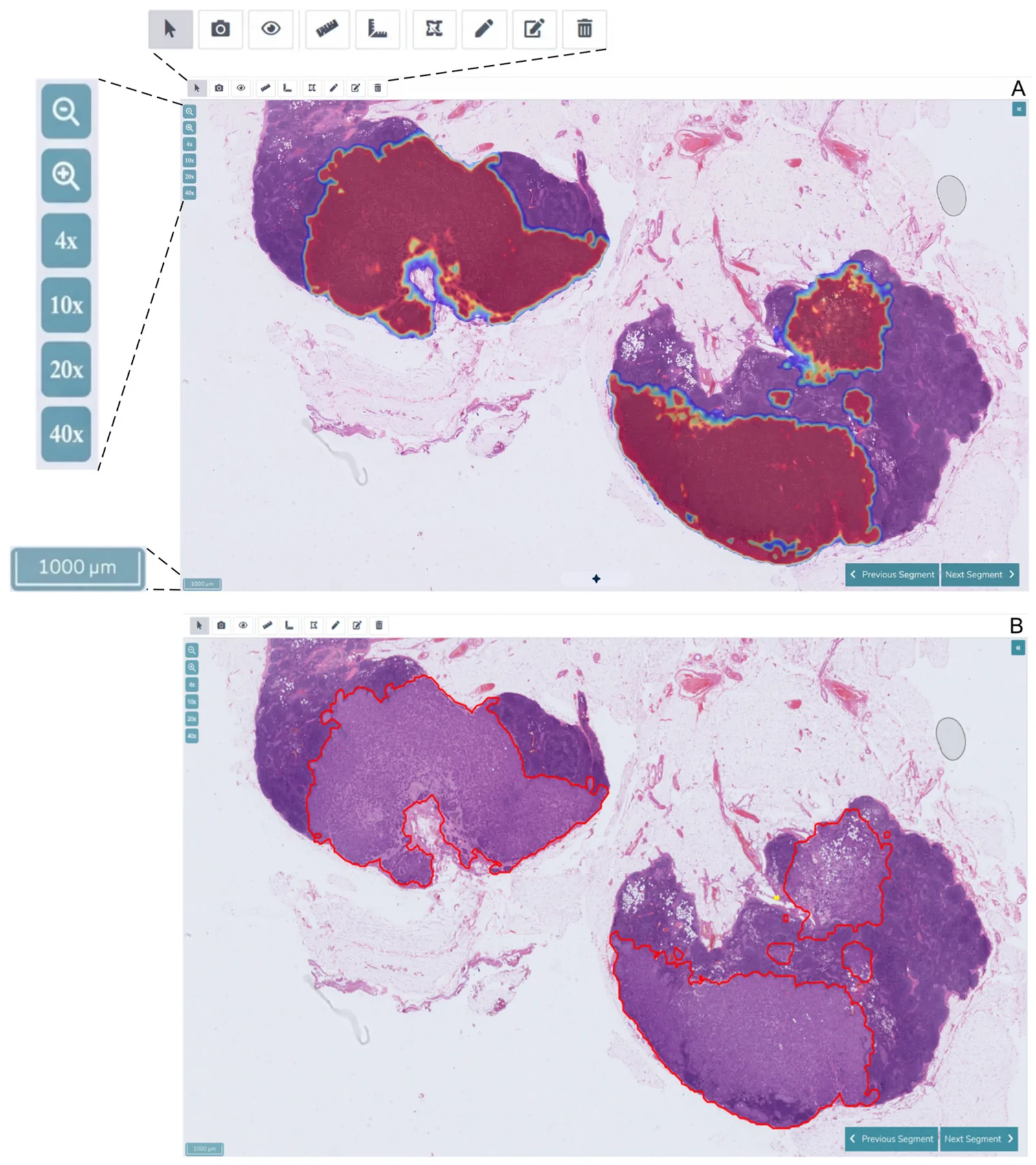
**Close-up views of LYDIA’s user interface**. (**A**) LYDIA’s heatmap annotation mode. (**B**) LYDIA’s region of interest annotation mode.

This study utilized two cohorts (Figure 2):

(a) A blinded external dataset for evaluation of LYDIA performance: The UMCU cohort comprised anonymized lymph node WSIs from metastatic breast, colon, lung, and skin tumors, collected retrospectively by the University Medical Center Utrecht (UMCU). Case-level tumor presence and cancer type were determined from initial reports (Supplementary Material Table S1). Images were uploaded to an AWS-based LYDIA deployment, with processing time and computational resources recorded. LYDIA generated a probabilistic heatmap of tumor presence, with a predefined cutoff (Figure 3). The study was approved by local institutional review boards.
(b) An internal reader performance study conducted in a simulated clinical workflow setting: Four histopathologists (>5 years of experience) evaluated 105 metastatic tumor WSIs (breast, colon, lung, skin) for metastatic status. The study spanned four weeks (February–March 2025), with pathologists assessing WSIs initially unaided and then assisted by LYDIA. WSIs were randomly shuffled and rotated between sessions to minimize memory bias. Diagnostic decisions and time-to-diagnosis were recorded for comparative analysis.

## 3. Model Training

### 3.1. Self-Supervised ViT-Based Feature Extraction with NeoPath

NeoPath (22M parameters) is a lightweight in-house Vision Transformer (ViT)-based Foundation Model trained using the DINO self-supervised learning framework [20]. Before training, the dataset was split at the patient level into training, validation, and test sets (70%, 10%, and 20%, respectively), followed by stratification based on tissue type. All WSIs from the same patient were assigned to a single subset to prevent data leakage between training, validation, and testing. For training NeoPath the whole model-training dataset was utilized.

During training, tissue regions were first identified using conventional image-analysis and color-based filtering to exclude background and non-tissue areas from patch sampling. Subsequently, 448 × 448-pixel patches were randomly extracted from the identified tissue regions using a mixture of 20× and 40× magnification. Sampling was performed according to the relative distribution of each tissue type in the training dataset, with approximately two million patches sampled per epoch.

The standard DINO augmentation strategy was applied, together with additional random zoom-in/zoom-out transformations and image noise [20]. These additional augmentations were introduced to increase variability in tissue scale and image appearance and to encourage the model to learn robust morphological representations rather than relying predominantly on color information. Training followed the original DINO optimization strategy and self-distillation loss [20], using an effective batch size of 256 patches. Briefly, the model was optimized using AdamW with an effective batch size of 256 patches, an initial learning rate of 5 × 10^−4^, and a 10-epoch linear warm-up followed by cosine learning-rate decay. Weight decay was increased from 0.04 to 0.4 following a cosine schedule, in accordance with the original DINO implementation [20]. NeoPath was trained for 100 epochs. Model performance was evaluated every five epochs using linear probing on the validation set, and the checkpoint with the highest validation performance was selected as the final model. The independent test set was used only for final evaluation.

### 3.2. Downstream Classification and Ensemble Prediction

Following self-supervised training, NeoPath was frozen and used as a feature extractor, generating a 384-dimensional embedding for each tissue patch. The extracted embeddings were subsequently used to train two complementary supervised classifiers: an XGBoost classifier [21] and a sparse convolutional neural network (CNN) [22] for detecting tumor presence on lymph nodes from the in-house and CAMELYON datasets.

The XGBoost classifier operated directly on the patch-level embeddings to estimate the probability of tumor presence. For the sparse CNN, the original spatial coordinates of each patch within the WSI were retained and combined with the corresponding 384-dimensional embeddings to reconstruct a sparse spatial representation of the tissue. This approach allowed the classifier to incorporate both the morphological information encoded by NeoPath and the spatial context between neighboring tissue regions.

Training annotations were independently performed by three experienced histopathologists. In cases of disagreement, the final annotation was determined by majority consensus. The downstream dataset was also partitioned at the patient level into independent training, validation, and test subsets, ensuring that WSIs originating from the same patient were restricted to a single subset and preventing patient-level data leakage.

During inference, tumor probabilities generated by the XGBoost and sparse CNN classifiers were combined using equal weighting (50:50). The resulting probabilities were mapped to their corresponding WSI coordinates to generate tumor probability heatmaps. The heatmaps were subsequently normalized and thresholded to generate regions of interest (ROIs), which were displayed to the pathologists through the LYDIA interface.

## 4. Statistical Analysis

Non-parametric Wilcoxon signed-rank test [23] was performed for all pairwise comparisons. A *p*-value of less than 0.05 is considered significant.

### 4.1. UMCU Cohort Performance Analysis

LYDIA’s performance was evaluated at both slide and segment levels. Processing time per WSI was measured in seconds. Slide-level metrics were determined by comparing the largest ground truth tumor area against the maximum predicted patch-level probability within the AI-identified ROI. For negative cases, the highest predicted probability across the WSIs’ patches was used. Segment-level metrics assessed the alignment between highlighted potential tumor regions by LYDIA and actual tumor areas on H&E slides annotated by pathologists. Mean ± Standard Deviation (SD) for Sensitivity and False Positives Per Square Millimeter (FP/mm^2^) were reported, noting the average lymph node area was approximately 60 mm^2^.

### 4.2. Analysis of Retrospective Multi-Reader Assessment of Diagnostic Efficiency

For the retrospective multi-reader assessment of diagnostic efficiency, median ± 95% CI and percentage time saved per WSI were reported. All metrics were calculated by comparing cases that were positive for metastasis against negative cases.

## 5. Results

### 5.1. Dataset Collection Analytics

**Figure 4:**
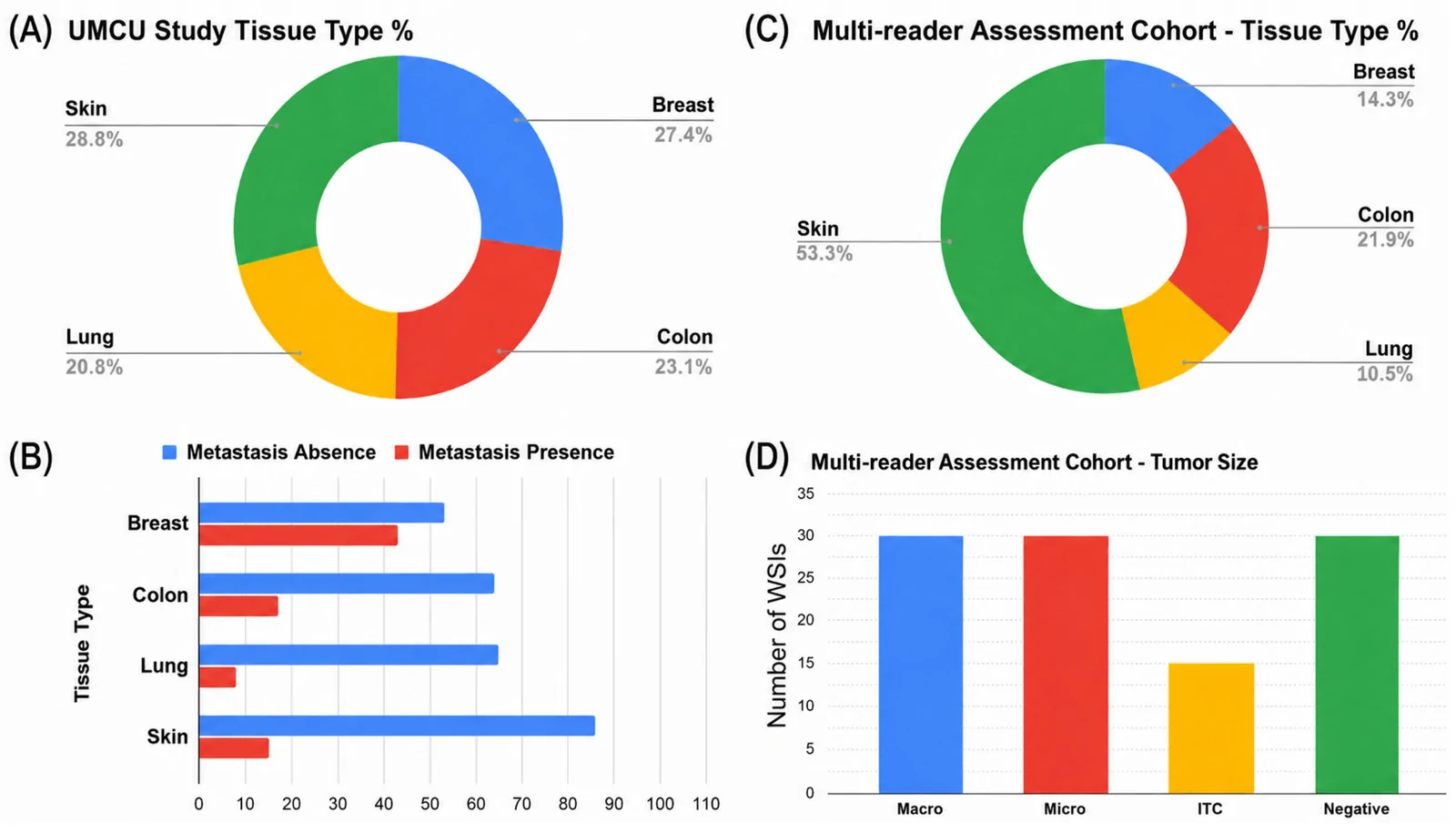
**Distribution of tissue types and metastasis categories across study cohorts.** (**A**) Proportional distribution of tissue types—breast, colon, lung, and skin—in the UMCU validation cohort (n = 351 WSIs); (**B**) case-level breakdown of benign versus malignant classifications across tissue types in the UMCU cohort; (**C**) tissue type distribution across sources in the retrospective multi-reader assessment cohort (n = 105 WSIs); (**D**) distribution of metastasis grading in the retrospective multi-reader assessment cohort, categorized as macro-metastasis, micro-metastasis, isolated tumor cells (ITCs), and negative cases.

We have curated two datasets for the two study cohorts (Table 2). The UMCU study cohort consists of 351 WSIs of lymph nodes derived from 39 anonymized cases including metastatic breast, colon, lung, and skin tumors. Patients’ demographics are present in Table 2. Initially, 15 WSIs from one skin case were excluded because it was a case of Warthin tumor (benign neoplasm). Tissue data distribution and percentage of tumor or benign cases per tissue type are present in Figure 4A,B. In addition to that, the retrospective multi-reader assessment cohort consists of 105 WSIs derived from the UMCU dataset. Details for the tissue distribution and metastasis grading are present in Figure 4C,D.

### 5.2. Evaluation of LYDIA’s Performance in Detecting Metastatic Tumors

We first evaluated the model’s performance in detecting metastatic tumors from breast, colon, lung tumors and skin in WSIs from lymph nodes, regardless of the tumor of origin, utilizing the UMCU study cohort. The ROC-AUC was highest for breast cancer (0.995), followed by skin (0.983), lung (0.973), and colon (0.963). Balanced accuracy (BA) at 99% sensitivity was 0.915 for breast, 0.938 for colon, 0.923 for lung, and 0.925 for skin. F1 scores were highest for breast cancer (0.905), then colon (0.800), skin (0.711), and lung (0.615). False positive rates (FPR) at this sensitivity threshold were lowest for skin (0.149) and highest for breast (0.170), with colon and lung at 0.123 and 0.154, respectively. True negative rates (TNR, specificity) were 0.830 for breast, 0.877 for colon, 0.846 for lung, and 0.851 for skin (Table 3).

LYDIA also exhibited robust segment-level performance across metastasis sizes and tissue types. For breast cancer, the model achieved perfect sensitivity (1.00 ± 0.00) for macro-metastases, maintained high sensitivity for micro-metastases (0.995 ± 0.025), and showed moderate detection of isolated tumor cells (ITCs) (0.757 ± 0.411), while maintaining low false positives per square millimeter (FP/mm^2^) values (0.071 ± 0.103). In colon cancer, sensitivity was perfect for macro- and micro-metastases (1.00 ± 0.00) but substantially lower for ITCs (0.600 ± 0.548), accompanied by slightly elevated FpSqMM (0.166 ± 0.639). Lung cancer segments exhibited perfect sensitivity for macro- and micro-metastases (1.00 ± 0.00) with acceptable ITC detection (0.667 ± 0.577), showing the highest FpSqMM (0.280 ± 0.478). Skin segments showed near-perfect sensitivity for macro-metastases (1.00 ± 0.00) and strong detection of micro-metastases (0.944 ± 0.192), with moderate ITC identification (0.625 ± 0.479), while maintaining the lowest FpSqMM values (0.048 ± 0.098) among all tissue types (Table 4).

### 5.3. Characterization of LYDIA Processing Time and Resources

**Figure 5:**
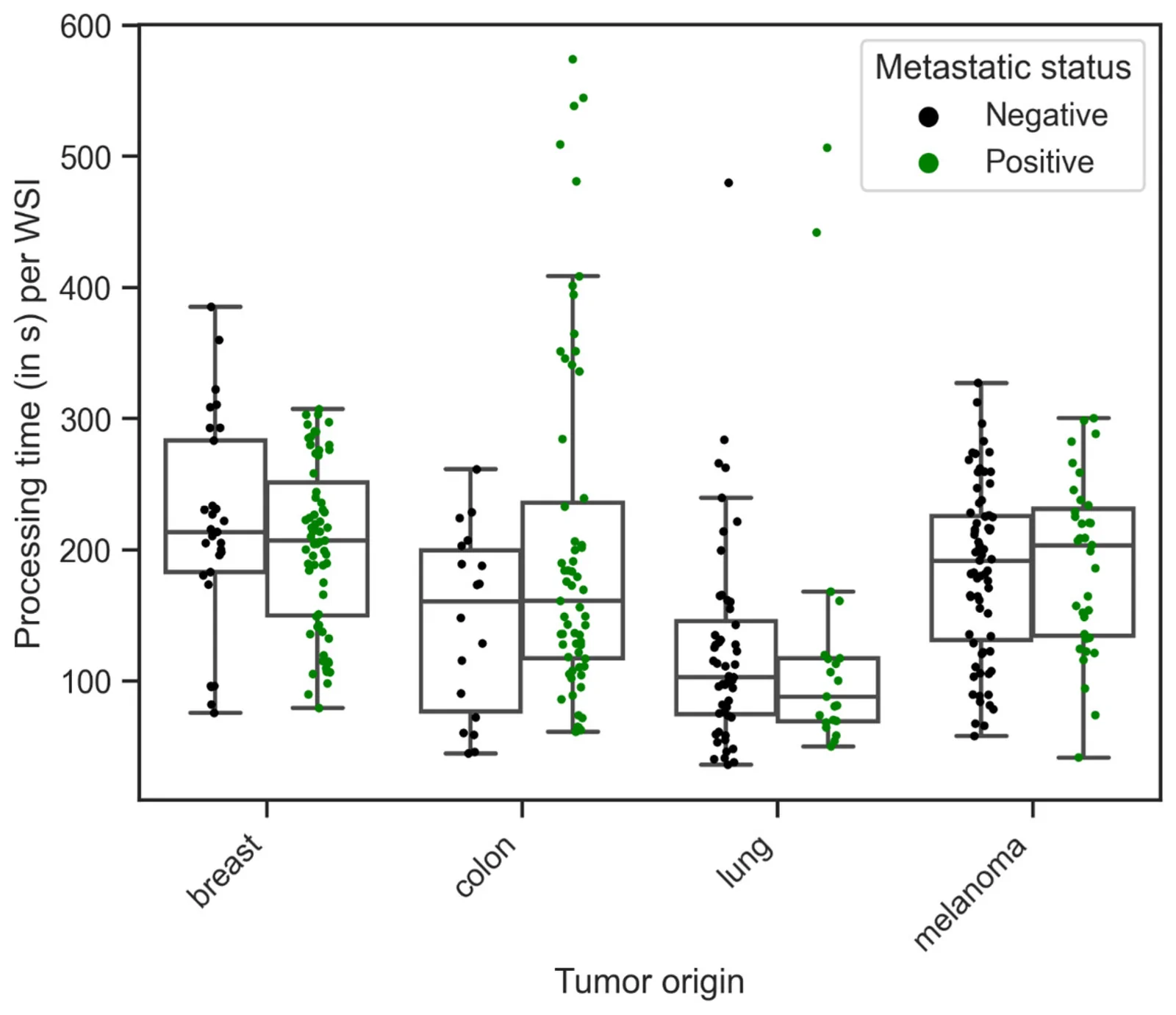
**LYDIA’s WSI processing times by tissue type and tumor status.** Average processing times (in seconds) for H&E-stained lymph node WSIs across breast, colon, lung, and skin cases, stratified by tumor status (positive vs. negative). Analysis was performed on AWS EC2 G4 instances equipped with NVIDIA T4 GPUs. Minimal variation was observed between positive and negative cases within each tissue type, and lung WSIs exhibited the shortest average processing times. The system’s architecture supports parallelized slide allocation across GPUs, enabling scalable performance for high-throughput diagnostic applications.

H&E-stained lymph node whole-slide images (WSIs) were processed using Amazon Web Services (AWS) EC2 G4 instances, featuring NVIDIA T4 GPUs and custom Intel Cascade Lake CPUs. The average processing time per WSI, irrespective of tumor origin, was approximately 3 min (180.73 ± 92.34 s). Granular analysis revealed consistent processing durations regardless of the tumor origin (Figure 5, Table S2).

### 5.4. Retrospective Multi-Reader Assessment of Diagnostic Efficiency

**Figure 6:**
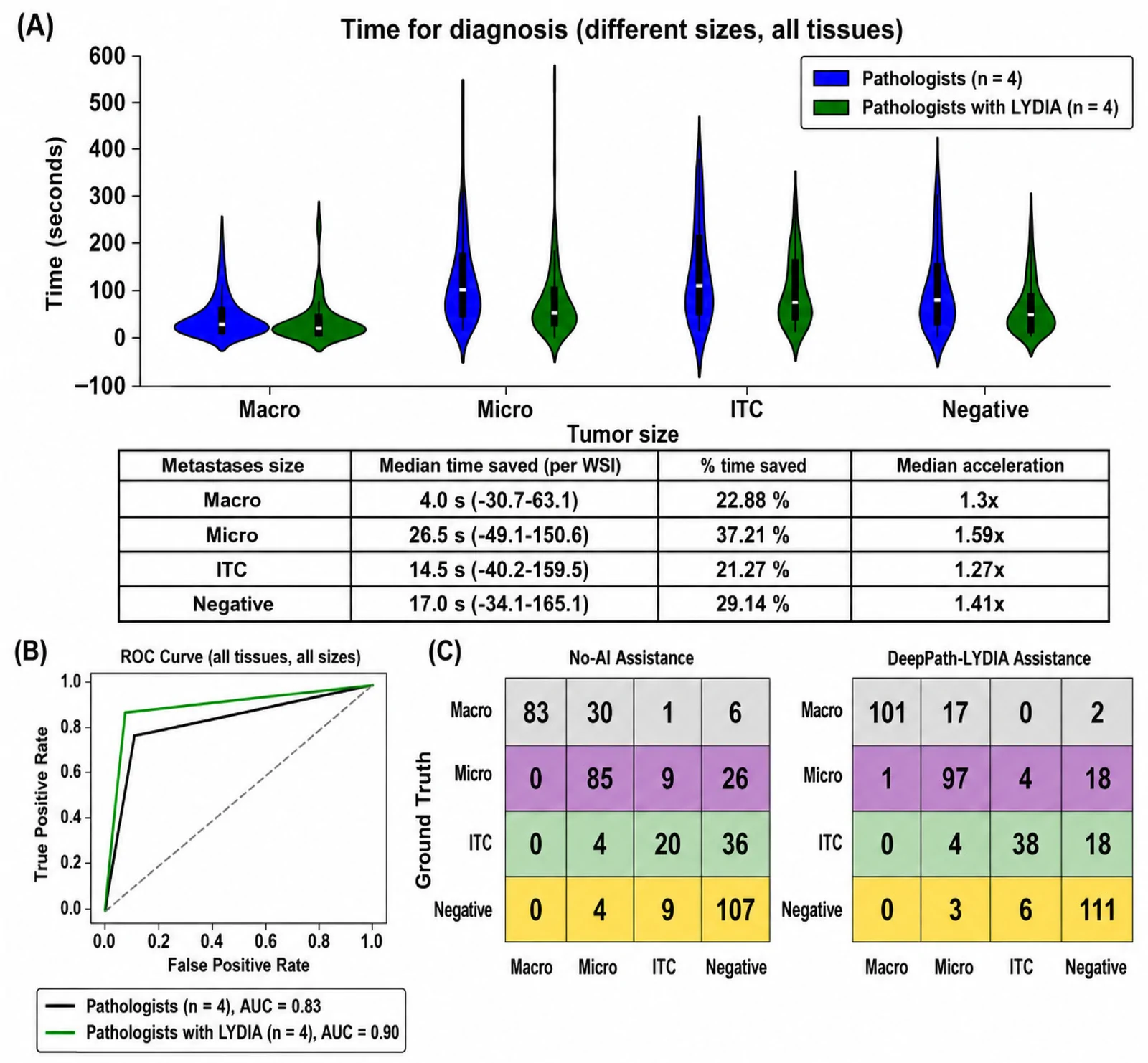
**Impact of LYDIA on diagnostic time and accuracy.** (**A**) Time-to-diagnosis per WSI for macro-metastases, micro-metastases, isolated tumor cells (ITCs), and negative cases, comparing AI-assisted and unassisted pathologist performance. Across all categories, AI assistance significantly reduced diagnostic time, with the largest time savings observed for micro-metastases; (**B**) improvement in diagnostic accuracy with LYDIA support. Receiver operating characteristic (ROC) AUC increased from 83% to 90%; (**C**) confusion matrices indicating pathologists’ diagnostic accuracy with and without AI assistance for Macro, Micro, ITC and Negative cases.

To evaluate LYDIA’s benefit as a decision support tool, 105 whole-slide images were independently assessed by four experienced histopathologists over four weeks (Table S3). AI-assisted diagnosis significantly reduced time-to-diagnosis across all categories from an average of 90.07 s per slide without AI-assistance to 60.72 s with AI-assistance (1.48-fold, *p* < 0.001). For macro-metastases, a 1.3-fold acceleration saved 4 s [95% CI: −30.7 to 63.1 s]. Micro-metastases saw a 1.59-fold acceleration, saving 26.5 s [95% CI: −49.1 to 150.6 s] per WSI. Isolated tumor cells were detected 1.27-fold faster, saving 14.5 s [95% CI: −40.2 to 159.5 s], and negative cases were processed 1.41-fold faster, saving 17 s [95% CI: −34.1 to 165.1 s] (Figure 6A).

Beyond time savings, LYDIA significantly enhanced pathologists’ diagnostic performance, increasing the area under the receiver operating characteristic curve (ROC-AUC) from 83% to 90% (Figure 6B). Across all sizes, the combined impact of LYDIA was notable: pathologists’ BA rose from 83.25% to 89.91%, F1 score from 85.13% to 91.76%, and true positive rate (TPR, sensitivity) from 77.33% to 87.33%. Concurrently, FPR decreased from 10.83% to 7.50%, FNR decreased from 22.67% to 12.67%, and true negative rate (TNR, specificity) improved from 89.17% to 92.50%. For macro-metastases, balanced accuracy (BA) improved from 92.08% to 95.42%, F1 score from 92.3% to 95.5%, and true positive rate (TPR, sensitivity) from 95.00% to 98.33%, and false positive rate (FPR) decreased from 10.83% to 7.50%. For micro-metastases, BA increased from 83.75% to 88.75%, F1 score from 82.8% to 88.3%, TPR from 78.33% to 85.00%, and false negative rate (FNR) decreased from 21.67% to 15.00%. The most significant improvement was in ITC detection, with BA rising from 64.58% to 81.25%, F1 score from 49.5% to 75.7%, TPR from 40.00% to 70.00%, and FNR halved from 60.00% to 30.00% (Table 5).

## 6. Discussion

The digitization of histopathology and the advent of AI, such as LYDIA, promise significant advancements in accelerating diagnosis while maintaining quality and reducing costs [24,25]. This study comprehensively evaluated LYDIA, an AI tool for detecting metastatic tumors in lymph node WSIs across breast, lung, colon, and skin cancers, via two scenarios: a blinded external validation (UMCU dataset) and a retrospective multi-reader assessment assessing real-world decision support diagnostic efficiency.

LYDIA demonstrated strong performance on the UMCU dataset, consistently detecting macro- and micro-metastases with high sensitivity. Detection of isolated tumor cells was more variable, reflecting the inherent difficulty of these subtle findings. False positive rates differed by tissue type, likely due to morphological complexity. In the retrospective multi-reader assessment of diagnostic efficiency, AI assistance significantly enhanced pathologist performance, improving diagnostic accuracy and consistency, particularly for micro-metastases and ITC, and notably reducing turnaround times. These findings align with prior studies demonstrating AI’s ability to reduce diagnostic turnaround times and improve consistency in challenging scenarios [9]. LYDIA’s strong performance likely stems from its use of advanced techniques, including a self-supervised Vision Transformer (ViT)-based foundation model (NeoPath), consistent with recent advancements in the field [26,27].

A critical distinction between LYDIA and earlier AI diagnostic models lies in its broad generalizability and underlying methodological architecture. While a competing system evaluated in the CONFIDENT-B [11] trial established the clinical utility of AI in breast cancer sentinel lymph nodes, LYDIA extends this utility across multiple primary tumor types. This generalizability was confirmed in a blinded, retrospective multi-reader study at University Medical Center Utrecht (UMCU) involving 455 patients across breast, colon, lung, melanoma, head and neck, and vulvar cancers [13]. In that study LYDIA was compared against the competing system of the CONFIDENT-B trial and presented an overall superior performance across cancer types and tumor sizes, which is also in accordance with the current study. It consistently detected macro- and micro-metastases with high sensitivity—100% for macro-metastases and 98.7% for micro-metastases—while detection of isolated tumor cells was more variable (77.4% sensitivity), reflecting the inherent difficulty of these subtle findings. The system maintained an acceptable average specificity of 7.6 false positive alerts per WSI, with false positive rates varying by tissue type, likely due to differences in morphological complexity. In the accompanying assessment of diagnostic efficiency, AI assistance significantly enhanced pathologist performance, improving diagnostic accuracy and consistency—particularly for micro-metastases and ITC—and notably reducing turnaround times, consistent with prior studies demonstrating AI’s ability to improve consistency and efficiency in challenging diagnostic scenarios [10]. LYDIA’s strong and generalizable performance stems from its use of advanced self-supervised Vision Transformer (ViT)-based foundation model architecture (NeoPath), consistent with recent advancements in the field [26,27].

The time savings observed in our study are comparable to those reported by [10] and the CONFIDENT-B trial [11], where substantial workflow efficiency gains with AI-assisted histopathological assessment were also demonstrated. More specifically in the CONFIDENT-B trial, AI support was associated with an estimated cost saving of approximately €15 per case due to reduced immunohistochemistry (IHC) utilization. As AI assistance enables pathologists to directly identify metastatic disease on initial hematoxylin–eosin (HE) slides, the need for follow-up IHC staining to rule out metastases is reduced. Given the comparable reductions in time-to-diagnosis observed across this study and the current one, the shared diagnostic mechanism, and LYDIA’s superior performance relative to the AI system used in CONFIDENT-B, it is reasonable to infer that similar reductions in IHC usage—and associated cost savings—can be expected in comparable clinical settings. Collectively, these findings highlight the potential of AI integration in routine pathology to improve diagnostic efficiency, optimize resource utilization, and enhance the sustainability of healthcare systems facing increasing diagnostic demands [28].

LYDIA supports scalable cloud deployment, analyzing each WSI in approximately 3 min on a single GPU, with parallel processing capabilities enabling efficient scale-up, limited only by available resources. This rapid, 3 min inference capability is particularly relevant for intraoperative applications. By providing near real-time diagnostic decision support, LYDIA could allow surgical teams to assess nodal status in a timely manner while the patient is still in the operating room, thereby directly informing immediate soft tissue flap reconstruction decisions. The model’s remarkable ROC-AUC across all tested cancer types demonstrates its universality, with potential for expansion to more metastatic tumor types. This independent evaluation of LYDIA confirms AI’s potential to accelerate diagnosis and increase precision in histopathology, aligning with previous findings [29]. Further follow-up studies and cost–benefit analyses are needed to quantify LYDIA’s full clinical advantage, particularly regarding its integration into real-time surgical pipelines for soft tissue repair.

## 7. Limitations and Future Directions

This study has several limitations. It was conducted using a single commercial AI system and involved a single institution with a limited group of pathologists for the clinical workflow evaluation, as well as a retrospective validation cohort, which may limit the generalizability of the findings. Future prospective, multi-center studies, along with systematic benchmarking across diverse AI solutions, are warranted to further validate and extend these results. Crucially, future collaborative research involving both pathology and reconstructive surgery departments is needed to prospectively evaluate LYDIA’s performance in real-time, intraoperative settings. Such studies should aim to quantify the direct clinical impact of AI-assisted nodal assessment on immediate soft tissue reconstruction rates, operating room time reduction, and long-term reconstructive outcomes.

This study has several limitations. It was conducted using a single commercial AI system and involved a single institution with a limited group of pathologists for the clinical workflow evaluation, as well as a retrospective validation cohort, which may limit the generalizability of the findings. In particular, the reliance on a single institutional setting means that potential sources of variability—such as differences in tissue processing protocols, scanner hardware, staining reagents, and pathologist experience across sites—were not captured, and these factors can meaningfully influence AI model performance in real-world deployment. Future prospective, multi-center studies are therefore essential to confirm the generalizability of LYDIA’s performance across diverse patient populations, laboratory workflows, and technical environments, and to validate its clinical utility under routine, real-world conditions. Such multi-center efforts should be complemented by systematic benchmarking against diverse AI solutions, allowing direct comparison of performance, usability, and clinical value across competing systems and further strengthening the evidence base needed to support broader adoption into clinical practice. Crucially, future collaborative research involving both pathology and reconstructive surgery departments is needed to prospectively evaluate LYDIA’s performance in real-time, intraoperative settings. Such studies should aim to quantify the direct clinical impact of AI-assisted nodal assessment on immediate soft tissue reconstruction rates, operating room time reduction, and long-term reconstructive outcomes. Finally, ongoing efforts to extend LYDIA’s functionality toward predicting the primary origin of cancers of unknown primary (CUP), a task shown to be feasible [30], represent a promising direction that warrants dedicated prospective validation, given its potential to further streamline diagnostic workup in this particularly challenging clinical scenario.

## 8. Conclusions

The integration of AI, specifically tools like LYDIA, represents a transformative shift in cancer diagnosis and subsequent surgical management. This study demonstrates LYDIA’s high efficiency and precision in analyzing lymph node WSIs for various cancer types, exhibiting robust diagnostic performance and workflow benefits. These findings suggest that future guidelines could formally recommend validated AI-powered decision support systems, such as LYDIA, as adjuncts to routine histopathological assessment of lymph nodes in cancer patients. By bridging the gap between pathology and the operating room, such rapid diagnostic systems can streamline intraoperative workflows, providing the timely nodal assessments necessary to guide safe, immediate soft tissue reconstruction. By reducing diagnostic turnaround time and unnecessary IHC staining (leading to direct cost savings) without compromising accuracy, AI-assisted workflows have the potential to increase diagnostic efficiency and optimize resource utilization. Reinvesting these saved resources into advanced soft tissue repair and bioengineering initiatives will ultimately foster a more comprehensive and sustainable continuum of care for oncology patients.

## Figures and Tables

**Table 1:** Dataset used for initial model training and validation.

Tumor Type	WSI Total	WSI Total %
Prostate	9999	65.01%
Cervix	585	3.80%
Breast	515	3.35%
Colon	372	2.42%
GI (apart from colon)	269	1.75%
Melanoma	243	1.58%
Lung	216	1.40%
Brain	100	0.65%
Other	3081	20.03%
**Total**	**15,380**	**100.00%**

**Table 2:** UMCU patients’ demographics.

Tissue Site	No. of Patients	Female	Male	Median Age Range	Age Range (Min–Max)
Colon	10	1	9	55–60	40–80
Skin	9	3	6	65–70	35–85
Breast	10	10	0	45–50	25–85
Lung	10	4	6	60–65	40–80

**Table 3:** LYDIA WSI-level performance characteristics at >99% sensitivity on the UMCU dataset.

	Breast	Colon	Lung	Skin
ROC-AUC (Sensitivity independent)	0.995 ± 0.020	0.963 ± 0.037	0.973 ± 0.037	0.983± 0.029
Balanced accuracy (BA)	0.915± 0.055	0.938 ± 0.048	0.923 ± 0.060	0.925 ± 0.059
F1 score	0.905 ± 0.058	0.8 ± 0.078	0.615 ± 0.110	0.711 ± 0.102
False positive rate (FPR)	0.17 ± 0.073	0.123 ± 0.064	0.154 ± 0.082	0.149 ± 0.080
True negative rate (TNR, Specificity)	0.83 ± 0.077	0.877 ± 0.064	0.846 ± 0.082	0.851 ± 0.080

**Table 4:** LYDIA segment-level performance characteristics.

	Sensitivity			
Tissue Type	Macro	Micro	ITC	FP/mm^2^
Breast	1 ± 0	0.995 ± 0.025	0.757 ± 0.411	0.071 ± 0.103
Colon	1 ± 0	1 ± 0	0.600 ± 0.548	0.166 ± 0.639
Lung	1 ± 0	1 ± 0	0.666 ± 0.577	0.280 ± 0.478
Skin	1 ± 0	0.944 ± 0.192	0.625 ± 0.479	0.048 ± 0.098

**Table 5:** Pathologists’ diagnostic performance with and without LYDIA assistance in retrospective multi-reader assessment of diagnostic efficiency.

	Pathologists	Pathologists + LYDIA	Pathologists	Pathologists + LYDIA	Pathologists	Pathologists + LYDIA	Pathologists	Pathologists + LYDIA
Tumor Detected	All Sizes		Macro-Metastasis		Micro-Metastasis		ITC	
Balanced accuracy (BA)	83.25 ± 4.2%	89.91 ± 3.4%	92.08 ± 4.3%	95.41 ± 3.3%	83.75 ± 7.2%	88.75 ± 6.1%	64.58 ± 13.3%	81.25 ± 10.8%
F1 score	85.13 ± 3.9%	91.76 ± 3.1%	92.30 ± 4.2%	95.54 ± 3.3%	82.81 ± 7.4%	88.31 ± 6.2%	49.48 ± 13.9%	75.67 ± 11.8%
True positive rate (TPR, Sensitivity)	77.33 ± 4.7%	87.33 ± 3.7%	95.00 ± 3.4%	98.33 ± 2.0%	78.33 ± 8.0%	85.00 ± 6.9%	40.00 ± 13.5%	70.00 ± 12.6%
False negative rate (FNR)	22.67 ± 4.7%	12.67 ± 3.7%	5.00 ± 3.4%	1.67 ± 0.5%	21.67 ± 8.0%	15.00 ± 6.9%	60.00 ± 13.5%	30.00 ± 12.6%

	**Pathologists**				**Pathologists + LYDIA**			
Tumor Free	**Negative slides**							
False positive rate (FPR)	10.83 ± 3.5%				7.50 ± 2.9%			
True negative rate (TNR, Specificity)	89.17 ± 3.5%				92.50 ± 2.9%			

## Data Availability

The datasets used in this study (the in-house dataset for model-training, the UMCU cohort and the retrospective multi-reader assessment cohort) contain sensitive clinical information. Due to ethical and privacy restrictions regarding patient data, the raw histopathology images cannot be made publicly available. Requests for access to the anonymized datasets for research purposes may be directed to the corresponding author and will be evaluated on a case-by-case basis, subject to institutional approval and data sharing agreements.

## Supplementary Materials

The following supporting information can be downloaded at: https://www.mdpi.com/article/10.3390/bioengineering13091021/s1, Figure S1: LYDIA correctly identifies a benign Warthin tumor in case Skin-0009; Table S1: Breakdown of metastasis subtype and WSI count per case in the UMCU validation cohort; Table S2: Mean processing times per WSIs for metastatic breast, colon, lung tumors and Skin; Table S3: Comparison of diagnostic assessment and time-to-diagnosis with and without AI assistance.
