# Supplementary material for: Evaluating the Performance of LYDIA: An AI-Powered Assistant in the Detection of Metastatic Tumors to Optimize Clinical Workflows and Inform Soft Tissue Surgical Decision-Making
Source: Bioengineering (Basel). 2026 Sep 1;13(9):1021. doi: 10.3390/bioengineering13091021 (PMC13603246; doi:10.3390/bioengineering13091021)
Supplement: Supplementary file 1 [file bioengineering-13-01021-s001.zip › bioengineering-4471107-supplementary.pdf]

Supplementary material

Supplementary Figure

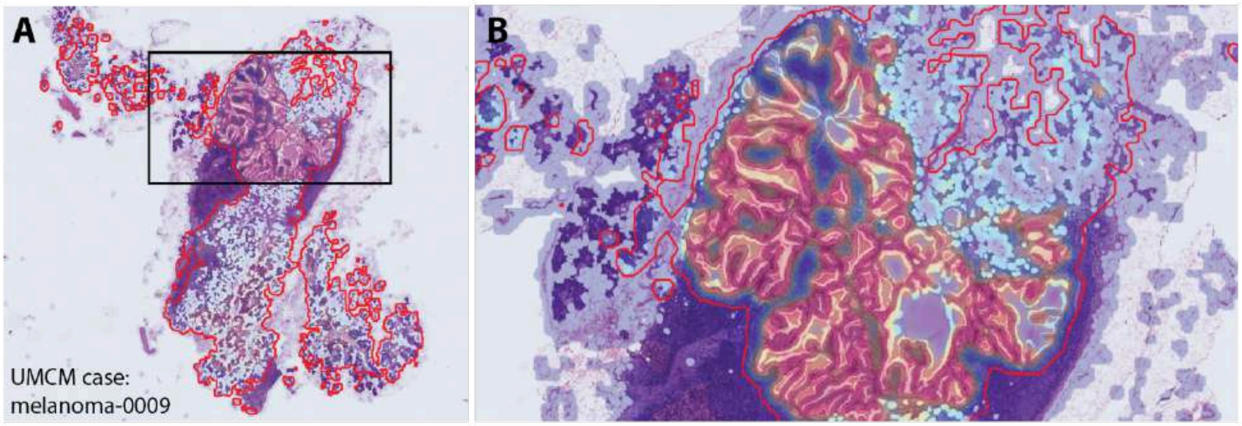

Figure S1: (A) Image panel displaying LYDIA's output for the Skin-0009 case from the UMCU, demonstrating LYDIA's ability to detect Warthin tumors (benign neoplasms); (B) Zoomed-in view of the region in which the neoplasm was detected, marked by the black box in (A).

Supplementary Tables

Table S1: Breakdown of metastasis subtype and WSI count per case in the UMCU validation cohort

Case	Cancer Type	Macro	Micro	ITC	Negative	Total
breast-0001	No tumor	0	0	0	5	5
breast-0002	Invasive carcinoma nst	10	0	0	5	15
breast-0003	Invasive carcinoma nst	0	0	0	5	5
breast-0004	Ducto-lobular carcinoma	0	0	0	5	5
breast-0005	Invasive carcinoma nst	0	0	0	5	5
breast-0006	No Tumor	0	0	0	6	6
breast-0007	Invasive carcinoma nst	5	0	0	5	10
breast-0008	Invasive lobular carcinoma	16	3	0	11	30
breast-0009	Invasive carcinoma nst	0	1	3	1	5

breast-0010	Invasive lobular carcinoma	4	1	0	5	10
colon-0001	Adenocarcinoma	0	1	1	8	10
colon-0002	Mucinous Adenocarcinoma	3	0	1	9	13
colon-0003	Mucinous Adenocarcinoma	0	0	0	1	1
colon-0004	Low-grade Adenocarcinoma	0	0	0	6	6
colon-0005	High-grade mucinous adenocarcinoma	8	0	0	19	27
colon-0006	Low-grade adenocarcinoma	1	0	0	5	6
colon-0007	Low-grade adenocarcinoma	0	0	0	3	3
colon-0008	Low-grade adenocarcinoma	1	1	0	5	7
colon-0009	Low-grade adenocarcinoma	0	0	0	7	7
colon-0010	No malignity	0	0	0	1	1

lung-0001	Typical carcinoid	2	0	0	5	7
lung-0002	No malignity	0	0	0	10	10
lung-0003	Solid adenocarcinoma	0	0	0	6	6
lung-0004	Lung adenocarcinoma	2	2	0	8	12
lung-0005	SCC	0	0	0	14	14
lung-0006	Metastasis carcinoma	2	0	0	0	2
lung-0007	Moderately differentiated acinar adenocarcinoma	0	0	0	3	3
lung-0008	Moderately differentiated mucous adenocarcinoma	0	0	0	6	6
lung-0009	Moderately differentiated lepidic adenocarcinoma	0	0	0	5	5
lung-0010	Moderately differentiated lepidic adenocarcinoma	0	0	0	8	8
Skin-0001	Invasive carcinoma NST	0	0	0	12	12

Skin-0002	Melanoma	0	0	0	12	12
Skin-0003	No tumor	0	0	0	6	6
Skin-0004	No tumor	0	0	0	6	6
Skin-0005	Melanoma	0	0	0	6	6
Skin-0006	No tumor	2	0	0	6	6
Skin-0007	No melanoma - nodular BCC	0	0	0	24	24
Skin-0008	Isolated tumor cells	1	0	0	7	8
Skin-0010	SCC metastasis	0	12	0	7	19

Table S2: Mean processing times per WSIs for metastatic breast, colon, lung tumors and Skin.

Tumor origin	Metastatic Status	Processing time
Breast	Total	208.06 ± 68.73 seconds
	Positive only	203.83 ± 63.14 seconds
	Negative only	217.81 ± 80.54 seconds
Colon	Total	192.05 ± 123.28 seconds
	Positive only	205.53 ± 132.05 seconds
	Negative only	144.85 ± 69.94 seconds
Lung	Total	125.09 ± 91.75 seconds
	Positive only	128.83 ± 119.57 seconds
	Negative only	123.59 ± 79.19 seconds
Skin	Total	185.66 ± 65.16 seconds
	Positive only	188.56 ± 65.54 seconds
	Negative only	184.23 ± 65.39 seconds

Table S3: Comparison of diagnostic assessment and time-to-diagnosis with and without AI assistance.

Pathologist	slide_id	duration_AI-ON (sec)	assesment_AI-ON	duration_AI-OFF (sec)	assesment_AI-OFF	GroundTruth
Path1	132	10	Macro	20	Macro	Macro
Path1	138	8	Macro	15	Macro	Macro
Path1	139	14	Macro	38	Macro	Macro
Path1	140	17	Macro	13	Macro	Macro
Path1	130	19	Macro	32	Macro	Macro
Path1	141	106	ITC	346	Negative	ITC
Path1	142	272	ITC	227	ITC	ITC
Path1	143	170	ITC	140	Negative	ITC
Path1	144	142	Micro	149	ITC	ITC
Path1	131	236	Negative	299	Negative	Negative
Path1	133	159	Negative	225	Negative	Negative
Path1	134	83	Negative	303	Negative	Negative
Path1	135	115	Negative	215	Negative	Negative
Path1	136	63	Negative	142	Negative	Negative
Path1	137	142	Negative	238	Negative	Negative
Path1	146	119	ITC	81	ITC	ITC
Path1	145	36	Negative	99	Negative	Negative
Path1	147	39	Negative	93	Negative	Negative
Path1	148	54	Negative	171	Negative	Negative
Path1	149	62	Negative	228	ITC	Negative
Path1	150	48	Negative	49	Negative	Negative

Path1	152	68	Negative	46	Negative	Negative
Path1	153	22	Negative	79	Negative	Negative
Path1	154	79	Negative	45	Negative	Negative
Path1	155	41	Negative	84	Negative	Negative
Path1	156	138	Negative	138	Negative	Negative
Path1	167	11	Macro	10	Macro	Macro
Path1	168	10	Macro	17	Macro	Macro
Path1	166	6	Macro	10	Macro	Macro
Path1	164	10	Macro	10	Macro	Macro
Path1	157	9	Macro	14	Macro	Macro
Path1	158	10	Macro	16	Macro	Macro
Path1	159	7	Macro	9	Macro	Macro
Path1	161	10	Macro	8	Macro	Macro
Path1	162	98	Macro	157	Negative	Macro
Path1	165	18	Macro	16	Macro	Macro
Path1	160	142	ITC	88	Negative	ITC
Path1	163	104	ITC	112	ITC	ITC
Path1	189	194	Micro	257	ITC	Micro
Path1	181	71	ITC	154	ITC	ITC
Path1	179	205	ITC	359	Negative	ITC
Path1	182	167	ITC	336	Micro	ITC
Path1	180	121	ITC	275	Negative	ITC
Path1	183	199	Negative	177	Negative	Negative
Path1	184	205	ITC	303	Negative	Negative

Path1	185	132	Negative	178	Negative	Negative
Path1	186	115	Micro	217	Negative	Negative
Path1	187	198	Negative	228	Negative	Negative
Path1	188	139	Negative	280	Negative	Negative
Path1	233	16	Macro	15	Macro	Macro
Path1	203	17	Macro	29	Macro	Macro
Path1	217	20	Macro	14	Macro	Macro
Path1	230	203	Micro	173	Micro	Macro
Path1	208	29	Macro	135	Micro	Macro
Path1	195	86	Macro	115	Macro	Macro
Path1	227	64	Macro	200	Micro	Macro
Path1	228	44	Macro	106	Macro	Macro
Path1	224	83	Micro	121	Micro	Macro
Path1	226	89	Micro	95	Micro	Macro
Path1	240	9	Macro	14	Macro	Macro
Path1	243	41	Macro	13	Macro	Macro
Path1	229	94	Macro	50	Macro	Macro
Path1	201	61	Micro	75	Micro	Macro
Path1	215	60	Micro	80	Micro	Macro
Path1	200	76	Micro	70	Micro	Micro
Path1	204	89	Micro	46	Micro	Micro
Path1	205	146	Micro	465	Micro	Micro
Path1	213	82	Micro	203	Micro	Micro
Path1	221	59	Micro	162	Micro	Micro

Path1	190	88	Micro	104	Micro	Micro
Path1	231	34	Micro	143	Micro	Micro
Path1	212	51	Micro	43	Micro	Micro
Path1	192	74	Micro	63	Micro	Micro
Path1	196	89	Micro	47	Micro	Micro
Path1	223	127	Micro	133	Micro	Micro
Path1	207	54	Micro	103	Micro	Micro
Path1	191	59	Micro	80	Micro	Micro
Path1	206	124	Micro	183	Micro	Micro
Path1	216	167	Micro	193	Micro	Micro
Path1	218	99	Micro	114	Micro	Micro
Path1	197	75	Micro	77	Micro	Micro
Path1	193	69	Micro	373	Micro	Micro
Path1	211	81	Micro	244	Micro	Micro
Path1	198	100	Micro	127	Micro	Micro
Path1	214	104	Micro	201	Micro	Micro
Path1	199	124	Micro	390	Micro	Micro
Path1	210	51	Micro	49	Micro	Micro
Path1	209	41	Micro	106	Micro	Micro
Path1	220	52	Micro	236	ITC	Micro
Path1	222	93	Micro	126	Micro	Micro
Path1	234	130	Micro	266	Negative	Micro
Path1	219	147	Micro	273	Negative	Micro
Path1	232	197	ITC	263	Negative	Micro

Path1	235	236	ITC	222	Negative	ITC
Path1	194	167	ITC	253	Negative	ITC
Path1	202	148	ITC	278	Negative	ITC
Path1	225	194	Negative	227	Negative	ITC
Path1	245	102	Negative	324	Negative	Negative
Path1	236	210	Negative	260	Negative	Negative
Path1	237	139	Negative	206	Negative	Negative
Path1	238	110	Negative	130	Negative	Negative
Path1	244	168	Negative	344	Negative	Negative
Path1	239	110	Negative	268	Negative	Negative
Path1	241	107	ITC	102	ITC	Negative
Path1	242	65	Negative	276	ITC	Negative
Path2	132	8	Macro	7	Macro	Macro
Path2	138	9	Macro	5	Macro	Macro
Path2	139	14	Macro	46	Negative	Macro
Path2	140	17	Macro	10	Macro	Macro
Path2	130	9	Macro	10	Macro	Macro
Path2	141	17	ITC	41	Negative	ITC
Path2	142	17	Negative	47	Negative	ITC
Path2	143	42	Negative	14	Negative	ITC
Path2	144	26	Negative	37	ITC	ITC
Path2	131	79	Negative	38	Negative	Negative
Path2	133	22	Negative	24	Negative	Negative
Path2	134	17	Negative	24	Negative	Negative

Path2	135	37	Negative	53	Negative	Negative
Path2	136	20	Negative	12	Negative	Negative
Path2	137	31	Negative	48	Negative	Negative
Path2	146	50	ITC	14	Negative	ITC
Path2	145	11	Negative	11	Negative	Negative
Path2	147	13	Negative	10	Negative	Negative
Path2	148	17	Negative	11	Negative	Negative
Path2	149	17	Negative	15	Negative	Negative
Path2	150	20	Negative	20	Negative	Negative
Path2	152	7	Negative	10	Negative	Negative
Path2	153	12	Negative	10	Negative	Negative
Path2	154	14	Negative	11	Negative	Negative
Path2	155	14	Negative	14	Negative	Negative
Path2	156	20	Negative	21	Negative	Negative
Path2	167	10	Macro	7	Macro	Macro
Path2	168	7	Macro	15	Macro	Macro
Path2	166	5	Macro	7	Macro	Macro
Path2	164	5	Macro	5	Macro	Macro
Path2	157	16	Macro	18	Macro	Macro
Path2	158	9	Macro	9	Macro	Macro
Path2	159	6	Macro	4	Macro	Macro
Path2	161	8	Macro	9	Macro	Macro
Path2	162	15	Macro	23	Negative	Macro
Path2	165	9	Macro	13	Macro	Macro

Path2	160	41	ITC	32	ITC	ITC
Path2	163	22	ITC	23	ITC	ITC
Path2	189	49	ITC	22	Negative	Micro
Path2	181	18	ITC	27	Negative	ITC
Path2	179	51	ITC	34	Negative	ITC
Path2	182	79	Negative	39	Negative	ITC
Path2	180	19	ITC	41	Negative	ITC
Path2	183	20	Negative	15	Negative	Negative
Path2	184	12	Negative	22	Negative	Negative
Path2	185	14	Negative	23	Negative	Negative
Path2	186	11	ITC	12	Negative	Negative
Path2	187	27	Negative	22	Negative	Negative
Path2	188	17	Negative	19	Negative	Negative
Path2	233	20	Macro	12	Macro	Macro
Path2	203	15	Macro	11	Macro	Macro
Path2	217	8	Macro	22	Macro	Macro
Path2	230	18	Macro	60	Micro	Macro
Path2	208	18	Macro	40	Negative	Macro
Path2	195	21	Macro	43	ITC	Macro
Path2	227	17	Macro	36	Micro	Macro
Path2	228	62	Negative	52	Negative	Macro
Path2	224	19	Micro	32	Micro	Macro
Path2	226	61	Negative	40	Micro	Macro
Path2	240	8	Macro	7	Macro	Macro

Path2	243	8	Macro	7	Macro	Macro
Path2	229	26	Macro	32	Micro	Macro
Path2	201	16	Micro	68	Micro	Macro
Path2	215	10	Micro	16	Micro	Macro
Path2	200	33	Negative	47	Negative	Micro
Path2	204	26	Negative	19	Micro	Micro
Path2	205	92	Negative	47	Micro	Micro
Path2	213	47	Negative	26	Negative	Micro
Path2	221	20	Micro	25	Micro	Micro
Path2	190	29	Micro	27	Micro	Micro
Path2	231	26	Micro	43	Micro	Micro
Path2	212	26	Micro	28	Micro	Micro
Path2	192	36	Negative	100	Micro	Micro
Path2	196	17	Micro	35	Micro	Micro
Path2	223	34	Negative	35	Negative	Micro
Path2	207	9	Micro	31	Micro	Micro
Path2	191	47	Negative	32	Micro	Micro
Path2	206	23	Negative	26	Negative	Micro
Path2	216	43	Negative	43	Negative	Micro
Path2	218	13	Negative	56	Negative	Micro
Path2	197	60	Micro	25	Micro	Micro
Path2	193	74	Micro	37	Micro	Micro
Path2	211	61	Negative	40	Negative	Micro
Path2	198	29	Micro	37	Micro	Micro

Path2	214	38	Micro	45	Micro	Micro
Path2	199	27	Negative	60	Negative	Micro
Path2	210	29	Micro	17	Negative	Micro
Path2	209	21	Micro	40	Micro	Micro
Path2	220	30	Micro	36	Negative	Micro
Path2	222	28	Micro	43	Negative	Micro
Path2	234	28	Negative	33	Negative	Micro
Path2	219	33	Negative	38	Negative	Micro
Path2	232	21	Negative	39	Negative	Micro
Path2	235	22	Negative	25	Negative	ITC
Path2	194	40	ITC	49	Negative	ITC
Path2	202	50	ITC	42	Negative	ITC
Path2	225	29	Negative	51	Negative	ITC
Path2	245	55	Negative	18	Negative	Negative
Path2	236	51	Negative	40	Negative	Negative
Path2	237	20	Negative	52	Negative	Negative
Path2	238	21	Negative	20	Negative	Negative
Path2	244	14	Negative	33	Negative	Negative
Path2	239	18	Negative	37	Negative	Negative
Path2	241	13	Negative	14	Negative	Negative
Path2	242	20	Negative	27	Negative	Negative
Path3	132	16	Macro	41	Macro	Macro
Path3	138	12	Macro	24	Macro	Macro
Path3	139	26	Macro	129	Macro	Macro

Path3	140	45	Macro	47	Macro	Macro
Path3	130	21	Macro	19	Macro	Macro
Path3	141	80	ITC	73	Micro	ITC
Path3	142	53	Negative	159	Negative	ITC
Path3	143	39	Micro	100	ITC	ITC
Path3	144	67	ITC	112	Negative	ITC
Path3	131	62	Negative	66	Micro	Negative
Path3	133	42	Negative	54	Micro	Negative
Path3	134	50	Negative	104	Negative	Negative
Path3	135	42	Negative	97	Negative	Negative
Path3	136	24	Negative	70	Negative	Negative
Path3	137	54	Negative	91	Micro	Negative
Path3	146	90	Negative	59	Negative	ITC
Path3	145	45	Negative	38	Negative	Negative
Path3	147	19	Negative	97	ITC	Negative
Path3	148	19	Negative	51	Negative	Negative
Path3	149	30	Negative	85	Negative	Negative
Path3	150	56	Negative	54	Negative	Negative
Path3	152	23	Negative	71	Negative	Negative
Path3	153	25	Negative	77	Negative	Negative
Path3	154	26	Negative	73	Negative	Negative
Path3	155	20	Negative	66	Negative	Negative
Path3	156	23	Negative	135	Negative	Negative
Path3	167	30	Macro	19	Macro	Macro

Path3	168	35	Macro	16	Macro	Macro
Path3	166	11	Macro	20	Macro	Macro
Path3	164	9	Macro	13	Macro	Macro
Path3	157	13	Macro	65	Macro	Macro
Path3	158	40	Macro	34	Macro	Macro
Path3	159	39	Macro	32	Macro	Macro
Path3	161	9	Macro	16	Macro	Macro
Path3	162	40	Micro	67	Negative	Macro
Path3	165	24	Macro	19	Macro	Macro
Path3	160	55	Negative	50	Negative	ITC
Path3	163	49	ITC	33	Negative	ITC
Path3	189	87	Micro	196	Negative	Micro
Path3	181	59	ITC	57	Negative	ITC
Path3	179	64	ITC	75	ITC	ITC
Path3	182	39	ITC	78	Negative	ITC
Path3	180	23	Micro	129	ITC	ITC
Path3	183	60	Negative	141	Negative	Negative
Path3	184	47	Negative	39	ITC	Negative
Path3	185	53	Negative	94	Negative	Negative
Path3	186	20	Micro	125	Negative	Negative
Path3	187	51	Negative	79	Negative	Negative
Path3	188	85	Negative	77	ITC	Negative
Path3	233	13	Macro	17	Macro	Macro
Path3	203	15	Macro	16	Macro	Macro

Path3	217	17	Macro	14	Macro	Macro
Path3	230	21	Macro	42	Micro	Macro
Path3	208	24	Macro	27	Micro	Macro
Path3	195	27	Macro	29	Micro	Macro
Path3	227	106	Micro	34	Micro	Macro
Path3	228	56	Macro	26	Micro	Macro
Path3	224	24	Macro	89	Micro	Macro
Path3	226	40	Macro	47	Micro	Macro
Path3	240	27	Macro	15	Macro	Macro
Path3	243	69	Macro	19	Macro	Macro
Path3	229	28	Macro	68	Micro	Macro
Path3	201	16	Micro	143	Micro	Macro
Path3	215	46	Micro	21	Micro	Macro
Path3	200	30	Macro	101	ITC	Micro
Path3	204	13	Micro	91	Micro	Micro
Path3	205	22	Micro	60	Micro	Micro
Path3	213	28	Micro	152	Micro	Micro
Path3	221	17	Micro	23	Micro	Micro
Path3	190	30	Micro	43	ITC	Micro
Path3	231	31	Micro	75	Micro	Micro
Path3	212	29	Micro	38	Micro	Micro
Path3	192	36	Micro	108	Micro	Micro
Path3	196	27	Micro	64	Micro	Micro
Path3	223	19	Micro	89	Micro	Micro

Path3	207	19	Micro	23	Micro	Micro
Path3	191	45	Micro	50	Micro	Micro
Path3	206	33	Micro	135	Micro	Micro
Path3	216	28	Micro	72	Micro	Micro
Path3	218	29	Micro	68	Negative	Micro
Path3	197	33	Micro	49	Micro	Micro
Path3	193	44	Micro	136	Micro	Micro
Path3	211	44	Micro	83	Micro	Micro
Path3	198	17	Micro	130	ITC	Micro
Path3	214	41	Micro	95	Micro	Micro
Path3	199	34	Micro	70	Micro	Micro
Path3	210	49	Micro	96	Negative	Micro
Path3	209	31	Micro	99	Micro	Micro
Path3	220	35	Micro	129	Micro	Micro
Path3	222	44	Micro	102	Negative	Micro
Path3	234	87	Negative	171	ITC	Micro
Path3	219	32	Micro	109	Negative	Micro
Path3	232	19	ITC	100	Negative	Micro
Path3	235	40	Negative	106	Negative	ITC
Path3	194	29	Micro	112	Negative	ITC
Path3	202	68	ITC	85	Micro	ITC
Path3	225	29	ITC	91	Negative	ITC
Path3	245	18	Negative	183	Negative	Negative
Path3	236	58	ITC	61	ITC	Negative

Path3	237	131	Micro	147	Negative	Negative
Path3	238	41	Negative	57	ITC	Negative
Path3	244	28	Negative	188	Negative	Negative
Path3	239	36	Negative	126	Negative	Negative
Path3	241	34	ITC	51	ITC	Negative
Path3	242	98	ITC	69	Negative	Negative
Path4	132	5	Macro	6	Macro	Macro
Path4	138	7	Macro	8	Macro	Macro
Path4	139	16	Macro	39	Macro	Macro
Path4	140	28	Macro	41	Macro	Macro
Path4	130	31	Macro	19	Macro	Macro
Path4	141	82	ITC	257	ITC	ITC
Path4	142	150	ITC	292	ITC	ITC
Path4	143	66	Negative	153	Negative	ITC
Path4	144	135	ITC	140	ITC	ITC
Path4	131	163	Negative	208	Negative	Negative
Path4	133	155	Negative	93	Negative	Negative
Path4	134	67	Negative	61	Negative	Negative
Path4	135	54	Negative	91	Negative	Negative
Path4	136	28	Negative	72	Negative	Negative
Path4	137	65	Negative	149	Negative	Negative
Path4	146	55	Negative	71	ITC	ITC
Path4	145	32	Negative	27	Negative	Negative
Path4	147	16	Negative	29	Negative	Negative

Path4	148	20	Negative	19	Negative	Negative
Path4	149	56	Negative	79	Negative	Negative
Path4	150	57	Negative	35	Negative	Negative
Path4	152	28	Negative	33	Negative	Negative
Path4	153	15	Negative	13	Negative	Negative
Path4	154	22	Negative	13	Negative	Negative
Path4	155	24	Negative	22	Negative	Negative
Path4	156	48	Negative	236	Negative	Negative
Path4	167	5	Macro	5	Macro	Macro
Path4	168	8	Macro	28	Macro	Macro
Path4	166	4	Macro	5	Macro	Macro
Path4	164	4	Macro	6	Macro	Macro
Path4	157	8	Macro	14	Macro	Macro
Path4	158	7	Macro	51	Macro	Macro
Path4	159	17	Macro	11	Macro	Macro
Path4	161	6	Macro	5	Macro	Macro
Path4	162	23	Macro	78	Macro	Macro
Path4	165	7	Macro	6	Macro	Macro
Path4	160	60	ITC	73	ITC	ITC
Path4	163	38	Negative	82	ITC	ITC
Path4	189	235	Negative	166	Negative	Micro
Path4	181	99	Negative	98	ITC	ITC
Path4	179	111	ITC	236	Negative	ITC
Path4	182	128	Negative	188	Negative	ITC

Path4	180	182	Negative	155	ITC	ITC
Path4	183	66	Negative	63	Negative	Negative
Path4	184	121	Negative	108	Negative	Negative
Path4	185	155	Negative	92	Negative	Negative
Path4	186	63	Negative	79	Micro	Negative
Path4	187	79	Negative	75	Negative	Negative
Path4	188	40	Negative	116	Negative	Negative
Path4	233	6	Macro	9	Macro	Macro
Path4	203	6	Macro	10	Macro	Macro
Path4	217	10	Macro	16	Macro	Macro
Path4	230	232	Macro	70	Macro	Macro
Path4	208	98	Macro	70	Macro	Macro
Path4	195	111	Macro	90	Micro	Macro
Path4	227	122	Macro	174	Micro	Macro
Path4	228	33	Macro	125	Micro	Macro
Path4	224	208	Micro	47	Micro	Macro
Path4	226	209	Micro	79	Macro	Macro
Path4	240	6	Macro	11	Macro	Macro
Path4	243	6	Macro	11	Macro	Macro
Path4	229	39	Micro	102	Macro	Macro
Path4	201	18	Micro	63	Micro	Macro
Path4	215	17	Micro	44	Micro	Macro
Path4	200	34	Micro	109	Micro	Micro
Path4	204	85	Micro	227	Micro	Micro

Path4	205	84	Micro	234	Micro	Micro
Path4	213	41	Micro	76	Micro	Micro
Path4	221	91	Micro	75	Micro	Micro
Path4	190	38	Micro	63	Micro	Micro
Path4	231	32	Micro	100	Micro	Micro
Path4	212	60	Micro	90	Micro	Micro
Path4	192	64	Micro	87	Micro	Micro
Path4	196	47	Micro	164	Micro	Micro
Path4	223	111	Micro	258	Micro	Micro
Path4	207	38	Micro	58	Micro	Micro
Path4	191	64	Micro	129	Micro	Micro
Path4	206	113	Micro	62	Micro	Micro
Path4	216	96	Micro	220	Micro	Micro
Path4	218	130	Micro	309	Micro	Micro
Path4	197	110	Micro	81	Micro	Micro
Path4	193	34	Micro	181	Micro	Micro
Path4	211	161	Micro	95	Micro	Micro
Path4	198	166	Micro	140	Micro	Micro
Path4	214	340	Micro	143	Micro	Micro
Path4	199	58	Micro	197	Micro	Micro
Path4	210	108	Micro	51	Micro	Micro
Path4	209	23	Micro	106	Micro	Micro
Path4	220	204	Micro	155	ITC	Micro
Path4	222	516	Micro	227	Micro	Micro

Path4	234	166	Micro	284	ITC	Micro
Path4	219	212	Negative	179	Negative	Micro
Path4	232	156	ITC	174	ITC	Micro
Path4	235	188	Negative	199	Micro	ITC
Path4	194	181	ITC	325	Negative	ITC
Path4	202	260	ITC	186	ITC	ITC
Path4	225	191	ITC	350	Negative	ITC
Path4	245	68	Negative	112	Negative	Negative
Path4	236	123	Negative	253	Negative	Negative
Path4	237	244	Negative	130	Negative	Negative
Path4	238	32	Negative	53	Negative	Negative
Path4	244	82	Negative	131	Negative	Negative
Path4	239	77	Negative	175	Negative	Negative
Path4	241	28	Negative	68	Negative	Negative
Path4	242	90	Negative	42	Negative	Negative
